# Note of Some Points in Regard to the Actions and Uses of Pilocarpin and Its Salts

**Published:** 1879-01

**Authors:** 


NOTE OF SOME POINTS IN REGARD TO THE ACTIONS AND USES OF PILOCARPIN AND ITS SALTS.—The alkaloid of Jaborandi, which is now known under the designation—Pilocarpin—is becoming plentiful enough to be employed in ordinary medical practice. Unfortunately the demand is not sufficient to justify its production on a large scale, hence the price continues very high. Having had an opportunity to use pilocarpin in a case to the treatment of which it is especially adapted, I concluded to test its action to compare the powers of the alkaloid with those of the crude drug. For this purpose I obtained some "Pilocarpin Muriatic, prepared by E. Merck, of Darmstadt, and imported by Mr. John Keeshan, of this city. As is well known, Merck has a very high reputation on the Continent for the purity of his preparations and chemicals.

The muriate of pilocarpin is a grayish white, crystalline, salt, freely soluble in water. I prepared a solution of two grains of the alkaloid to a half ounce of water—hence, ten minims represent one-twelfth of a grain, which I found to be a sufficient quantity for administration to an adult.

The physiological actions of jaborandi have been so exhaustively studied, that it seemed unnecessary to go further in testing the effects of its alkaloid, than merely to determine how nearly the two correspond. I have done so by some experiments *in propia persona.* I injected one-sixth of a grain of the muriate, hypodermically, and began to experience the characteristic effects in five minutes. A transient giddiness, flushing of the face, and frontal headache were the first symptoms experienced. A profuse salivary discharge followed—so profuse that a constant expectoration was necessary to keep the mouth free. Sweating came on soon after the salivation, appearing first on the forehead and thence over the body generally. It became so profuse in a short time as to saturate my undergarments. I next experienced a severe pain along the urethra and some irritability of the bladder, the urine becoming pale and watery without being increased in amount.

In five minutes the pulse had risen to 90 and was rather small and weak.

Feeling very uncomfortable and depressed, I concluded to arrest the action of the pilocarpin, and accordingly injected hypodermically 1-10 grain of atropia sulphate, the physiological antagonist. Nothing could be more exact than the counterbalancing action of these two remedies. In two minutes the skin which was wet and cold had become dry and warm, and the free salivary flow was replaced by a dry mouth; the headache disappeared, and the irritability of the bladder ceased.

My experiment demonstrated the genuineness and the physiological activity of pilocarpin. Its ready solubility, slight taste, and small dose render it most eligible for administration by the stomach or by the integument. For these reasons, and for the more important reason that the preparations of jaborandi are much sophisticated, the alkaloid should take the place of the crude drug in medical practice.

The application of jaborandi in the treatment of disease is an admirable illustration of the way in which a knowledge of the physiological actions of a remedy indicates its remedical employment. There has been no history of its empirical use, except vague traditions derived from savages; but when the actions became known, at once the range of its uses was marked out from the physiological stand-point.

Acting so powerfully on the skin, jaborandi is indicated in all conditions of diseases in which sweating is beneficial. Various instances have already been reported of its successful use in albuminuria, in renal dropsy, scarlatinal dropsy, and in serous effusions. In cardiac dropsy, although very beneficial, it must be used with caution, as it has sometimes caused irregular and fluttering action of the heart, with faintness. When such an untoward result is threatened or occurs, the free use of alcoholic stimulants is proper, and their administration has been successful under these circumstances. In one case of cardiac dropsy in my own practice, in which there was considerable præcordial oppression, the 1-16 of a grain caused a decided increase in the cardiac distress without any action on the salivary glands and skin. The result has been observed by others—notably by Dennue—who witnessed vomiting and depression, without the proper physiological action on the skin and salivary glands. Again, in other cases of cardiac dropsy, the results have been good and the heart unfavorably effected.

Recently I have had good results from pilocarpin in winter cough, (dry catarrh of the bronchi). In such a case great relief is always afforded by a remedy which increases the bronchial mucus and diminishes the viscidity of the exudation. When such cases are accompanied by a dry skin, by a scaly eruption, prurigo, or chronic urticaria—a combination quite usual in old subjects—the relief obtained by pilocarpin is really remarkable.

It will be found, no doubt, that jaborandi or its alkaloid will afford great relief in chronic cutaneous maladies characterized by diminished secretion of the sudoriporus glands. Influenced by the same considerations, it has already been employed in mumps, parotitis from ordinary causes, and in deficient mammary secretion.

Finally, I have observed that jaborandi has decided aphrodisiac qualities, and have used the fluid extract with success in functional impotence.

The readers of this journal have had placed before them very recently, the most important applications of pilocarpin in the treatment of eye diseases.

"Pilocarpin" says Dannue, "surpasses in promptness all other methods for obtaining abundant diaphoresis, and is less dangerous than hot baths. As the injection can be repeated several times daily without losing their effect, an enormous amount of transudation can be made to disappear rapidly in urgent cases."—*Cincinnati Lancet and Clinic.*

